# Safe delivery kits and newborn infection in rural Ethiopian communities

**DOI:** 10.3389/fpubh.2024.1305255

**Published:** 2024-08-09

**Authors:** Won Ju Hwang, Tae Hwa Lee

**Affiliations:** ^1^East-West Nursing Research Institute, College of Nursing Science Kyung Hee University, Seoul, Republic of Korea; ^2^School of Nursing, Yonsei University, Seoul, Republic of Korea

**Keywords:** cross-sectional observational research, Ethiopia, infection, newborn, safe delivery kit, supply kits

## Abstract

**Objectives:**

Our goal in this study to investigate the impacts of using safe delivery kits, along with education on their appropriate use, has on preventing newborn and maternal infection.

**Design:**

A cross-sectional study.

**Setting:**

Participants, and Interventions: we conducted the study on 23 sites across a rural district in Oromia Region, Ethiopia. Safe delivery kits were distributed by health extension workers. Participants comprised 534 mothers between the ages of 17 and 45 years, who were given a safe delivery kit at 7 months’ pregnancy for use during their subsequent delivery. Data collection was performed by the trained interviewers in rural Ethiopian communities.

**Results:**

Multiple logistic regression analyses showed an independent association between using the cord tie provided in the kits and decreased newborn infection. Specifically, newborns whose mothers used the cord tie were 30 times less likely to develop cord infection than those not using the cord tie in the kits. Further, mothers who received education regarding safe delivery kit use had lower rates of puerperal infection.

**Conclusion:**

Single-use delivery kits, when combined with education regarding the appropriate means of using the kit, can decrease the likelihood of maternal infection.

**Implications for nursing:**

Nurses and health extension workers in low and middle-income countries should educate mothers on safe delivery kits by providing information regarding their usefulness and the importance of correct and consistent use. Implications for Health Policy: our findings emphasize the need for further interventions in vulnerable countries designed to increase the rate of hygienic birthing practices for deliveries outside health-care facilities.

## 1 Introduction

Globally, 2.3 million children died in the first 20 days of life in 2022. There are approximately 6,500 newborn deaths every day, amounting to 47% of all child deaths under the age of 5 years (1).

Newborns have a higher mortality rate than children of older ages (2). The leading causes of newborn death include infection, suffocation during delivery, and complications related to premature birth. In particular, over half of all newborn mortalities are related to delivery environments (3, 4). Meanwhile, over 90% of maternal mortalities that occur in low and middle-income countries (LMIC) are caused by hemorrhage, infection, unsafe abortion, and eclampsia, and over 20% of such mortalities can be prevented by providing safe delivery environments (5).

In other words, the deaths of many newborns and mothers could be prevented by providing a hygienic delivery environment and trained assistants; unfortunately, the international community does not prioritize maternal-newborn health. Nevertheless, in countries in Sub-Saharan Africa, including Ethiopia, the mortality rate of children under 5 years of age has steadily decreased over the last two decades, and this has been attributed to international involvement; however, the mortality rate in this region remains higher than that in other countries. Despite the overall decrease in mortality among children younger than 5 years, the proportion of deaths that occur in the neonatal period is increasing (5, 6).

Furthermore, Ethiopia has made significant progress in reducing childhood mortality rates, but it still faces challenges. Ethiopia is among the top 10 countries with the highest neonatal mortality rate in 2020. Approximately, 97,000 babies die every year in their first 4 weeks of life in Ethiopia. Subnational neonatal morality and hospital-level neonatal mortalities are variable, particularly in developing or pastoralist regions; data are not readily available (7).

In Ethiopia, 94% of children are birthed at home unattended by trained persons. The government introduced an innovative strategy, the Health Services Extension Program, in 2003. Safe delivery service is a component of the program’s maternal and child health care package. However, little is known about the status of the service uptake. This study thus aimed to assess the utilization of clean and safe delivery service and associated factors in rural Ethiopia (7). Further, as of 2008, less than half of the Millennium Development Goals (MDGs) (8), which were targets for 2015 agreed by all United Nations member states, had been accomplished (5, 9). In particular, studies have reported that countries in Africa and Southeast Asia have not shown any improvement. Thus, health-care-related aid should focus on the Maternal and Child Health Project, which is one of the priority projects of the MDGs and SDG (10, 11). Safe delivery improves the health of both the mother and the newborn and, therefore, the health of the family, which is the fundamental element of economic development; thus, creating a safe delivery environment is important.

Infection is the main cause of infant and maternal mortality in low and middle-income countries (11). Infection in the first week of life is associated with maternal infection (12). While family planning interventions, high-quality delivery, and postpartum care, delivering in facilities are research-proven strategies to prevent maternal and neonatal mortality, many women in low and middle-income countries lack access to these interventions, even though such measures have been recommended as effective strategies by which governments can enhance maternal and child health-care services (13, 14). In low and middle-income countries such as Ethiopia, newborn infection is a primary cause of newborn death. However, prematurity and perinatal events (asphyxia) are also leading causes (14, 15). In general, newborn infection is caused by using contaminated delivery instruments in unhygienic delivery environments. Therefore, the most direct and fundamental solution is to control bacterial infection by providing a clean delivery environment.

There are only a few studies on water, sanitation, and hygiene (WASH) looking at this. We believe instruments and environmental containments play a large role in maternal and newborn infection, however, this is complicated. There are several issues to consider such as, mothers may not deliver in a facility which increases risk, they may have prolonged rupture of membranes. Prematurity and perinatal events (asphyxia) are also leading causes of newborn and maternal death (15).

The World Health Organization (WHO) has emphasized the need for six “clean” elements (clean hands, clean vagina, clean umbilical-cord-cutting instruments, clean cord-tying instruments, clean delivery surface, and clean cutting surface) to ensure a hygienic delivery environment (16). To help achieve such an environment, delivery kits, typically reusable, are given to community-based women and traditional birth attendants (TBAs) after training. The items in a safe delivery kit (SDK) include a plastic sheet for cleaning the delivery space, soap for the delivery helper, a surgical blade for cutting the umbilical cord, a clean cord tie, and a clamp for tying the umbilical cord with cotton. SDKs are also cost-effective (17, 18). Thus, the WHO states that this delivery kit is the most simple and useful tool for a clean delivery. The WHO has also begun to provide countries with a manual to enable them to customize the kit to suit the respective situations in their countries. A feasibility study reported that by distributing SDKs to rural regions with poor medical care facilities and high infant mortality rates, this project can decrease the risk of infection for newborns by nearly 80% (19). Also, it can contribute to the development of local communities, as residents can continue to operate the project by themselves. However, the meta-analysis has controversial outcomes (12).

We expand on previous research and conclusions about safe home delivery kits, including in Ethiopia and regionally. As TBAs are not part of the health system in Ethiopia formally, and health extension workers (HEWs) are, this study does not consider TBAs. HEWs provide prenatal/postnatal care and refer to skilled attendants. The family planning intervention service program is being implemented by deploying female HEWs who received training. Each Kebele will have a health post, the operational center for the HEWs (20). Antenatal care and clean and safe delivery services are among the elements of the maternal and child health services package (21). Implementing these elements focuses on empowering women, their families, and communities to recognize pregnancy-related risks and take responsibility for developing and implementing appropriate responses.

In order to solve infant and maternal mortality-related problems in low and middle-income countries, international organizations and many other non-government organizations (NGOs) are actively promoting the SDK as a cost-effective measure (5). For example, the United Nations Development Programme has successfully promoted “mama kits” in rural regions of Uganda (3); as a result, medical-specialist-supported deliveries have increased nine-fold, and cases of bacterial infection in the vagina, eye infection in newborns, diarrhea, tetanus, and sepsis, all of which could occur during delivery in an unhygienic environment, have fallen remarkably. In Pakistan, SDKs were distributed after providing TBAs with basic knowledge regarding delivery (21). Examinations consequently found that rates of maternal mortality and stillbirth decreased by almost 30%, and infection in newborns decreased by nearly 80% (22, 23). Thus, it is clear that the SDK project can create conditions for pregnant women to deliver their babies safely, mainly because most of the female residents in the targeted regions deliver babies at home or in an unhygienic environment (24).

### 1.1 Aim of the study

The purpose of the present study is to investigate the impacts of using SDK that is provided with education training on how to use the kit to prevent newborn and maternal infection.

## 2 Methods

### 2.1 Research design

This study utilizes data from community-based interventions designed to reduce maternal and newborn mortality conducted in 23 sites across a rural district of the Oromia region, which is situated in eastern Ethiopia. SDKs were distributed by health extension workers (HEWs) who received training in SDK use.

### 2.2 Sample and setting

In total, 599 female respondents to a pre-survey on a family planning intervention project. Of those, 534 mothers received and used a SDK during delivery. The women between 17 and 45 years of age were given SDKs by the trained personnel at 7 months of pregnancy. 89% of those who received SDK used them for subsequent deliveries. Data collection was performed by the trained interviewers in Woreda, Ethiopia, from October 22 to November 21, 2011.

### 2.3 Contents of the safe delivery kit

**Figure 1:**
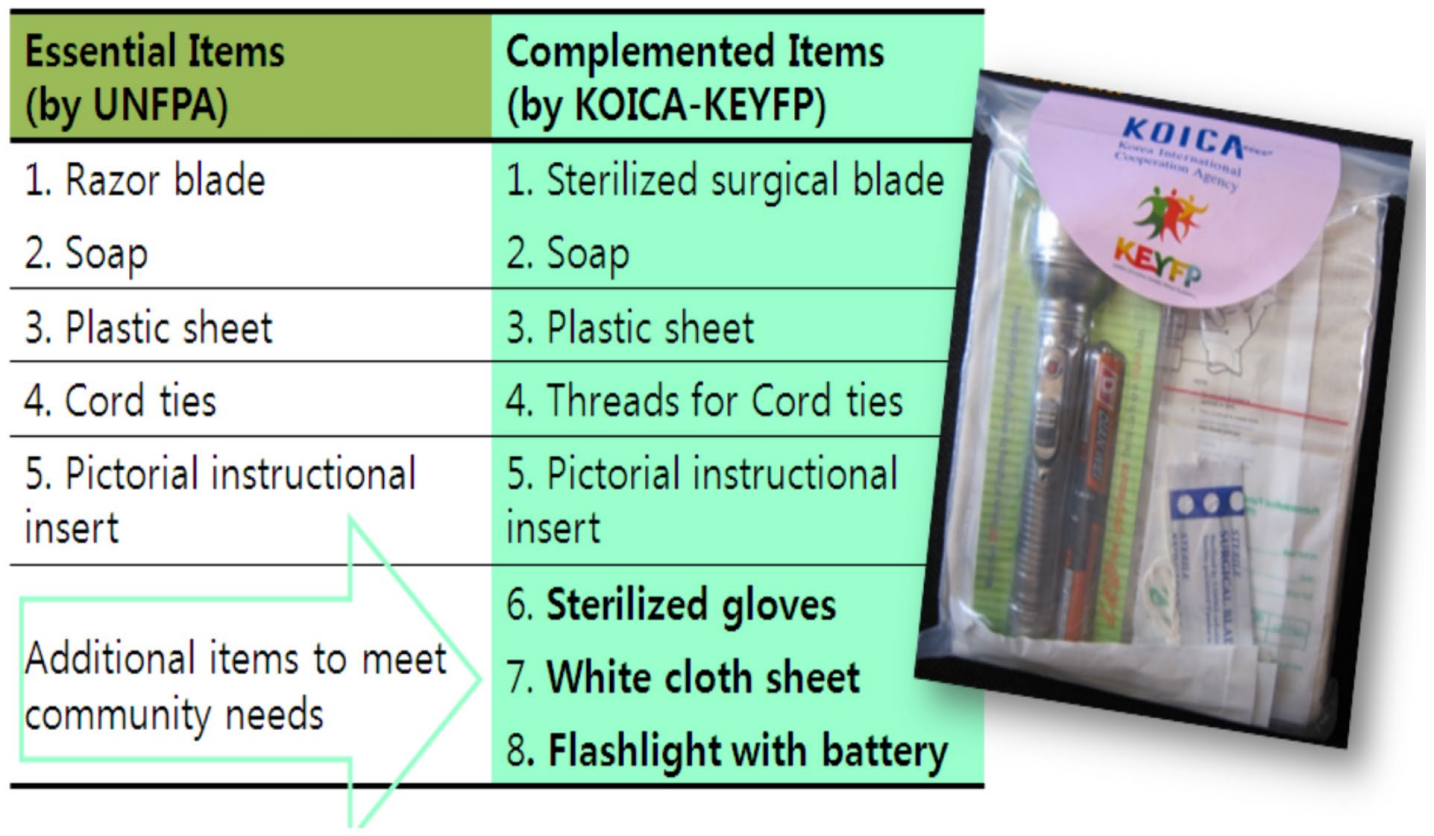
Safety delivery kit items.

Local health-care providers were consulted to determine the items that would be necessary for inclusion in an SDK for their respective communities (Figure 1). Consequently, the items selected for the kits were a surgical blade, soap, a cord tie, a plastic sheet, a fabric cover, a flashlight, a pair of sterilized gloves, and a user manual according to the need assessment.

### 2.4 Preparation, production, and promotion of SDK

**Figure 2:**
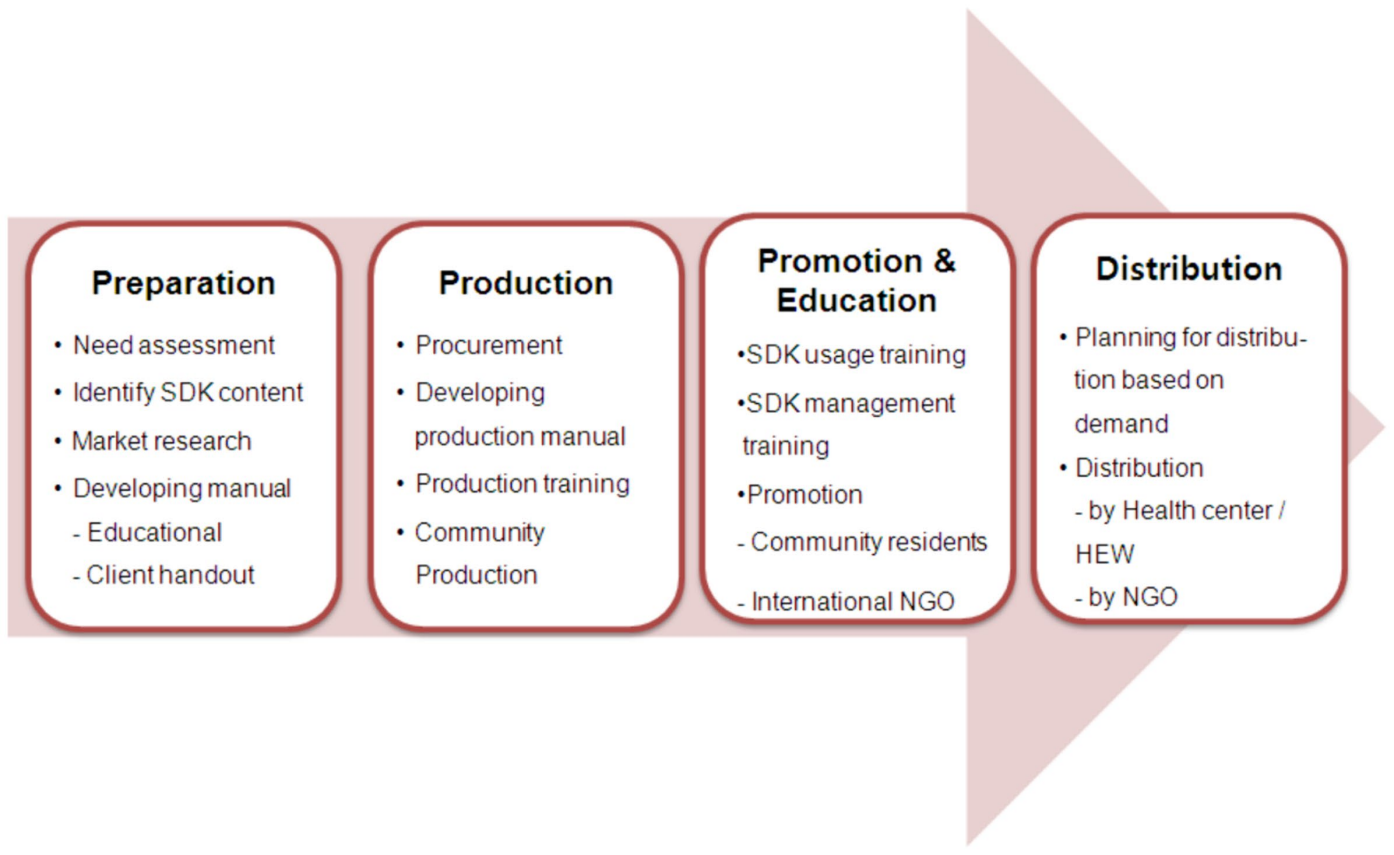
Safety delivery kit project procedure.

The SDK project comprised four phases (Figure 2). Phase 1 was the preparation phase. Each community’s opinions were collected by performing a needs assessment on the requirements of the community; then, after deciding on the items that should be included in the SDKs, such as soap, cord tie, and lantern, market research was conducted, the seven items included and a user’s manual was developed.

In order to collect opinions on means of producing and distributing SDKs in the community, a meeting with local residents was held, and meetings with parties related to the community were held on nine occasions across the 23 sites. Previously, to collect opinions on the means of producing and distributing SDKs in the community, one meeting with local residents was held in each community, and nine meetings with parties related to the community.

Phase 2 was the production phase. A production manual for the SDKs was developed, and local residents were educated and trained. Then, SDKs were produced by employing local female laborers. By November 2010, 11 local laborers had produced 15,000 SDKs over 59 days. The average daily production was 254 kits, and average production per person was 23 kits.

Phase 3 was promotion and education. Before distributing the SDKs, the residents of each community were given education regarding safe delivery and how the kits should be used. In order to distribute the SDKs efficiently, operation, management, and promotion training was provided. In the distribution phase, a distribution plan was established and implemented through need assessment. This included research such as the need assessment of pregnant women in the community. In order to increase local residents’ awareness of SDKs and to encourage them to effectively distribute and manage SDKs, various promotions were conducted for local leaders, medical specialists, and community leaders. Specifically, during the project period, a total of 57 SDK promotions were provided to a total of 19,662 community people. These promotions were performed at the Public Health Training Center which is located in or in communities in Oromia State. SDK promotions were held, with a total of 1,911 people in attendance. The promotion team collaborated with the local health center to hold over 22 program events for residents in the community, which had 4,098 attendees. Further, 16 promotions and campaigns were performed in collaboration with external centers. Additionally, 12 promotions were performed through outreach strategies, which 9,557 people attended.

### 2.5 Instruments

Survey instruments were developed by revising and supplementing a survey instrument for family planning and maternal-child health, a basic delivery kit guide, and a survey instrument testing satisfaction. This survey instrument was developed in English by the research team based on population survey and then revised after a review by a local adviser.

The questionnaire comprised six sections – the socioeconomic characteristics of the population, maternal-child health (delivery process, information regarding delivery helpers, etc.), use of SDKs, satisfaction with SDKs, distribution of SDKs, and infection rate of newborns and mothers.

### 2.6 Ethical approval

The study was conducted after obtaining approval from the Institutional Review Board (IRB) of the Yonsei University College of Nursing. For the face-to-face interviews, we employed well-trained survey interviewers with experience conducting national surveys with households in the community. Before the start of the survey, the interviewers were provided with a half-day training to familiarize them with the questionnaire, and a feedback meeting was held to assess how data should be collected. Comments were provided on aspects that needed improvement. The survey supervisor was responsible for the coordination and supervision of the overall data collection. Data quality control was performed by the supervisor who randomly cross-checked 10% of the questionnaires every day. Survey interviewers (one man and one woman working as a team).

A parent was required to sign a consent form for mothers under 19 years of age. All of the participants provided written informed consent to participate in this study, which the IRB also approved. In addition, all respondents provided written consent using paper consent forms, which were documented in the electronic data system. The relevant IRBs approved these consent processes.

### 2.7 Data collection

In locations where there were few female residents, surveys were performed through survey interviewers. The survey interviewer verbally explained the study objectives to each female resident, and an informed consent statement was then signed by the women. Meanwhile, in regions with many female residents, surveys were conducted with the cooperation of health centers and people based in Woreda. For illiterate women, the information sheet was read aloud. For some questions, data from the mother’s registration record was used to collect information on mothers before conducting the survey. Maternal and newborn puerperal infection was defined as the mother and newborn presenting with a fever.

### 2.8 Data analysis

Analysis of the data focused on measuring the impact SDK use had on puerperal infection in mothers and newborns (puerperal infection was defined as both the mother and newborn presenting with a fever). To measure this, multiple logistic regression analyses were performed This paper reports summary statistics on use of birth kits in the target population, and uses hierarchical logistic regression to analyze associations between baseline characteristics of respondents and use of birth kits, as well as associations between use of birth kits and outcomes (puerperal infection in mothers and newborns) during pregnancy and delivery.

## 3 Results

### 3.1 Characteristics of the study participants

The sociological characteristics of the mothers who reside in Hetosa Woreda are shown in Table 1. A total of 534 women participated in this survey, and their average age was 25.6 years. The respondents had been pregnant four times on average; almost 36.2% had more than five children, and most were married. Regarding religion, 396 (74.4%) were Muslim, 129 (24.2%) were Ethiopian Catholic, and seven (1.4%) were atheists or believers of other religions. Their education levels were very low: 230 (43.1%) were uneducated, 284 (53.2%) had graduated from elementary school, and only 20 (3.7%) had graduated from middle/high school. Most participants were married (95%) and had elementary-school-level education or lower (96%). The mean age was 26.6 years. Most participants delivered at home (94%) without a skilled birth attendant present (89%). Cord tie used by 519(96.1%) mothers in SDK. Only 35% used the user’s manual in the SDK (Table 2).

**Table 1:** Items used in the safe delivery kit.

Type	Mothers (*n* = 534)	
	*N*	%
Soap	519	(97.2)
Cord tie	513	(96.1)
Plastic sheet	519	(97.2)
Clothes sheet	521	(97.6)
Flashlight	471	(88.2)
Gloves	513	(96.1)
Surgical blade	506	(94.8)
Illustrated instruction manual	187	(35.0)

**Table 2:** Sociodemographic and gynecological characteristics of the participants.

Type		Mother (*n* = 534)	
		*N*	%
Age^*^	Average^†^	25.6 ± 4.43	
	15–19	18	(3.4)
	20–29	407	(76.9)
	30–39	100	(18.9)
	40–49	4	(0.8)
Marital status	Married	507	(95.1)
	Single	26	(4.9)
Number of children	0	4	(0.8)
	1–2	185	(34.7)
	3–4	151	(28.3)
	5+	193	(36.2)
	Average^†^	3.7 ± 2.15	
Number of pregnancies	1–2	170	(31.9)
	3–4	136	(25.5)
	5+	227	(42.6)
	Average^†^	4.1 ± 2.39	
Education	None	230	(43.1)
	Elementary school	284	(53.2)
	Middle/High school	20	(3.7)
Religion	Orthodox	129	(24.2)
	Muslim	396	(74.4)
	No religion/other	7	(1.4)

^†^Value is mean ± standard deviation. ^*^ indicates results influenced by missing values.

### 3.2 Factors influencing puerperal infection

Multiple logistic regression analyses showed an independent association between the use of the cord tie in the SDK and decreased newborn infection [OR = 0.03; 95% confidence interval (95%CI): 0.001–0.616; (Table 3)]. Mothers who received education regarding how to use the SDK had lower rates of puerperal infection (OR = 0.46, 95%CI: 0.24–0.88). Further, mothers who were assisted by HEWs and skilled birth attendants also had lower rates of infection (OR = 0.53, 95%CI: 0.28–1.02), although the statistical strength of this association was of borderline significance (*p* = 0.058).

**Table 3:** Results of multiple logistic regression on newborn infection.

Variables	*B*	Wald	df	*P*	Odds	95% CI	
						Lower	Upper
Age	0.036	0.036	1	0.38	1.04	0.96	1.12
Skilled birth attendant	−0.632	−0.632	1	0.06	0.53	0.53	1.02
Cord tie use	−0.907	−0.907	1	0.49	0.89	0.89	5.35
SDK training^*^	0.776	0.776	1	<0.05	0.46	0.46	0.88

^*^Only significant variables are shown. 95%CI: 95% confidence interval.

## 4 Discussion

Safe delivery kits are not a new phenomenon, having been widely distributed and marketed for a number of decades. However, the evidence underpinning this ‘commonsense’ intervention is not as robust as might be expected. Increasing the uptake of interventions that are effective for improving maternal and perinatal outcomes is critical. Supplying SDKs has been suggested as a feasible strategy for ensuring the timely availability and effective follow-up of care (5, 24).

Distribution and use of SDKs varies considerably from country to country, however most reports indicated some degree of health system involvement. The experience in Ethiopia suggests that SDKs can be harnessed to improve care, such as attendance at antenatal care. In contrast it has been suggested that the availability of a trained attendant using a kit may be a factor in encouraging ‘high-risk’ mothers to give birth at home rather than travel to a health center (23, 24). More data are urgently needed before making recommendations to further scale up mother held birth kits or to expand kit contents.

The importance of context cannot be over-emphasized, and better descriptive methods are needed to capture contextual factors that may impact the implementation process. For example, by using local laborers for production, the capacity of the residents was strengthened, and new means of income generation were created for this community. To move this work forward, a multidisciplinary approach, the SDK, is essential.

### 4.1 Findings

The present study aimed to measure whether promoting an SDK project can decrease infection in mothers and newborns and, ultimately, lower maternal and child mortality rates in rural areas of Ethiopia, where health-care service for mothers and newborns is poor. Through various promotional activities and education, community residents’ awareness of safe delivery was improved, and community health-care providers were educated regarding how to use and manage SDKs. Additionally, a community capacity building program was created, which enabled local residents to continue to operate the project. By using local laborers for production, the competence of the local residents was strengthened and a new means of income generation was created for this community.

The key findings of the present study are that most births in Ethiopia occur at home and that the use of the SDK was not widespread. SDKs have been distributed in Ethiopia since about 2010. It may not have saturated this region, but multiple international NGOs have been involved with the kits donated by the United Nations Population Fund (UNFPA) (25). The study also found that education regarding the SDK was a significant determinant of puerperal infection in mothers. It is clear from the present results that when the SDK-provided cord tie is used during delivery, newborns are significantly less likely to have umbilical infections.

The result of the systematic review shows that the SDK can decrease the infection rate in newborns and mothers in rural areas of Ethiopia indicating a similar level of effectiveness as that reported in a previous meta-analysis of the effect of such kits (2), and can be tailored to suit the delivery environment and residents’ needs in the region. A total of 15,000 SDKs were produced and distributed to almost 90% of the 16,000 pregnant women in the project region; also, an education benefit was provided to the pregnant women who received an SDK. Thus, most pregnant women in the project region received important benefits as a result of using the safe-delivery-related services.

Second, educational and promotional activities targeted toward local residents and medical-care providers improved awareness of safe delivery, demonstrated the importance of using SDKs, and increased the accessibility of safe delivery services for residents of rural areas. Promotions of the SDKs also strengthened community competence. As a result, this project, involving producing and distributing SDKs, successfully contributed to decreasing infection rates in newborns in rural areas of Ethiopia. Studies have shown that health-care providers, such as midwives and birth attendants, can prevent approximately two-thirds of the deaths among women and newborns, provided they are well-trained, well-equipped, well-supported, and authorized (24, 26). Considering the fact that few women in the study region deliver their babies at hospital facilities, instead delivering at home with the help of family members (because of local traditional delivery customs), it is necessary to expand infrastructure for health-care services and encourage women to deliver babies at hospitals with professional delivery assistants, which would ultimately decrease infant and maternal mortality (14, 27).

### 4.2 Study limitations

There are some limitations to this research. First, this was a cross-sectional study rather than a pre-post study, and we did not use an objective measure of puerperal infection; instead, we tested for infection by checking the infant for fever. Therefore, it is possible that some participants were missed. Second, other organizations also provided health education with SDK in the area. Data was collected by trained personnel, who were different from HEWs, who also educated the participants.

Finally, fever was the most experienced danger sign in our study and in most of the other studies reviewed, except in Africa, where fever was frequently represented as an infection sign (12, 25). This is because fever is more easily identified than other neonatal infection signs (28). However, we only have early studies on we only have early studies on water, sanitation, and hygiene (WASH) looking at this (28). We believe instruments and environmental containments play a large role in driving maternal and newborn sepsis, but it is complicated. Mothers may not deliver in a facility, which is an increased risk, may have prolonged rupture of membranes, etc.

## 5 Conclusion

These findings emphasize the need for further interventions for increasing the rate of hygienic birthing practices for deliveries occurring outside facilities (29). Several meta-analyses on SDKs, including this study (21), have been conducted. SDKs are well-proven in-home or unattended deliveries.

Notably, the present results support the existing theory that it is important to use the cord tie included in the SDKs (21) and also shows that mothers can and should be trained to use such kits properly (30, 31). It is reported that, in order to lower postpartum infections, it is not sufficient to merely train birth attendants (32–34); greater emphasis must be placed on the use of these kits by mothers, especially during home births (35). Further, it is important not only to distribute SDKs, but also to provide training regarding their usefulness and the importance of their correct and consistent use.

### 5.1 Implications for nursing

Nurses, midwives, and health extension workers in low and middle-income countries should educate mothers on SDKs by providing information regarding their usefulness and the importance of their correct and consistent use.

### 5.2 Implications for health policy

Our findings emphasize the need for further interventions for increasing the rate of hygienic birthing practices in vulnerable countries in regard to deliveries that occur outside health-care facilities.

### 5.3 Implications for practice and/or policy

1. What is already known about this topic? (include key points and/or knowledge gaps).
  All newborns are exposed to a higher mortality risk compared to children of older ages. Many newborn deaths can be prevented by providing a hygienic delivery environment and trained helpers.
2. What this paper adds: (research findings/key new information).
  Newborns whose mothers used the cord tie in the delivery kit were 30 times less likely to develop cord infection. When combined with education about Safe Delivery Kits, single-use delivery kits can decrease the likelihood of maternal infection.
  The implications of this paper: (how findings influence or can be used to change policy/practice/research/education).

Medical professionals and health workers should educate mothers on the proper use of Safe Delivery Kits by providing information about their usefulness and the importance of their correct and consistent use. The findings emphasize the need for further interventions aimed at increasing the rate of hygienic birthing practices for deliveries occurring outside health care facilities.

## Data availability statement

The raw data supporting the conclusions of this article will be made available by the authors, without undue reservation.

## Ethics statement

The studies involving humans were approved by Yonsei University institutional review board. The studies were conducted in accordance with the local legislation and institutional requirements. The participants provided their written informed consent to participate in this study.

## Author contributions

WH: Conceptualization, Writing – original draft, Writing – review & editing. TL: Funding acquisition, Project administration, Supervision, Writing – original draft.
